# Supplementary material for: Pharmacologic and genetic evidence converge on mechanisms of psychotic illness
Source: Transl Psychiatry. 2025 Jul 23;15:254. doi: 10.1038/s41398-025-03456-7 (PMC12287348; doi:10.1038/s41398-025-03456-7)
Supplement: Supplementary file 1 — Supplementary Tables Captions [file 41398_2025_3456_MOESM1_ESM.docx]

**Supplementary Table Captions**

**Supplementary Table 1**

List of the 64 antipsychotic drugs defined using the Anatomical Therapeutic Chemical Classification (ATC) Level 3.

**Supplementary Table 2**

List of the 124 Medical Dictionary for Regulatory Activities (MedDRA) terms used to represent psychotic illness.

**Supplementary Table 3**

List of the 276 propsychotic drugs defined using Vigibase.

**Supplementary Table 4**

List of 66 adverse events associated with the propsychotic drugs in Supplementary Table 3.

**Supplementary Table 5**

The proportion of propsychotics mapping to each of 85 different pharmacological subgroups defined by ATC Level 3.

**Supplementary Table 6**

Results of analyses linking propsychotics and antipsychotics to target genes.

**Supplementary Table 7**

Results of analyses linking propsychotics and antipsychotics to mechanisms of action on target genes.

**Supplementary Table 8**

Table of targets showing the observed proportion and p-value for upregulated and downregulated mechanisms of action.
